# Palmitic Acid and Oleic Acid Differently Modulate TLR2-Mediated Inflammatory Responses in Microglia and Macrophages

**DOI:** 10.1007/s12035-022-02756-z

**Published:** 2022-01-25

**Authors:** Anne-Marie Howe, Sinéad Burke, Marcella E. O’Reilly, Fiona C. McGillicuddy, Derek A. Costello

**Affiliations:** 1 UCD School of Biomolecular & Biomedical Science, University College Dublin, Dublin 4, Ireland; 2 UCD Conway Institute, University College Dublin, Dublin 4, Ireland; 3 UCD School of Medicine, University College Dublin, Dublin 4, Ireland

**Keywords:** Palmitic acid, Oleic acid, Diet-induced obesity, Nitric oxide, Cytokine, Microglia

## Abstract

The relationship between systemic immunity and neuroinflammation is widely recognised. Infiltration of peripheral immune cells to the CNS during certain chronic inflammatory states contributes significantly to neuropathology. Obesity and its co-morbidities are primary risk factors for neuroinflammatory and neurodegenerative conditions, including Alzheimer’s disease (AD). Dietary fats are among the most proinflammatory components of the obesogenic diet and play a prominent role in the low-grade systemic inflammation associated with the obese state. Saturated fatty acid (SFA) is largely implicated in the negative consequences of obesity, while the health benefits of monounsaturated fatty acid (MUFA) are widely acknowledged. The current study sought to explore whether SFA and MUFA differently modulate inflammatory responses in the brain, compared with peripheral immune cells. Moreover, we assessed the neuroinflammatory impact of high-fat-induced obesity and hypothesised that a MUFA-rich diet might mitigate inflammation despite obesogenic conditions. Toll-like receptor (TLR)2 mediates the inflammation associated with both obesity and AD. Using the TLR2 agonist lipoteichoic acid (LTA), we report that pre-exposure to either palmitic acid (PA) or oleic acid (OA) attenuated cytokine secretion from microglia, but heightened sensitivity to nitric oxide (NO) production. The reduction in cytokine secretion was mirrored in LTA-stimulated macrophages following exposure to PA only, while effects on NO were restricted to OA, highlighting important cell-specific differences. An obesogenic diet over 12 weeks did not induce prominent inflammatory changes in either cortex or hippocampus, irrespective of fat composition. However, we reveal a clear disparity in the effects of MUFA under obesogenic and non-obesogenic conditions.

## Introduction

Neuroinflammation is the specific response that occurs within the central nervous system (CNS) in response to damage or infection. The degree of neuroinflammation is influenced by a variety of factors, including the duration and the source of the insult [1]. Acute inflammation comprises the immediate and early response against an infectious agent and is the first line of defence which aims to remove the insult and repair the damage. In contrast, chronic inflammation results from a more persistent inflammatory stimulus and is integral to the pathogenesis of CNS disease [2]. Neuroinflammation underlies various CNS conditions, including those of psychiatric nature such as anxiety [3, 4] and depression [5, 6]. In addition, it is central to the pathology of neurodegenerative diseases such as Parkinson’s disease (PD) [7, 8] and Alzheimer’s disease (AD) [9]. This is generally understood to result from dysregulation of microglial activation, favouring a chronic production of proinflammatory mediators that promote pathological changes and neurobehavioural complications such as depression and cognitive deficits [10]. Moreover, chronically activated cells can disrupt the integrity of the blood–brain barrier (BBB), leading to infiltration of systemic immune cells and mediators, which further exacerbates the proinflammatory environment in the CNS [11].

The impact of inflammatory insults on neuronal and cognitive dysfunction has been widely reported, and we among others have illustrated the key role of microglial Toll-like receptors (TLRs) in mediating these effects [12–17]. In recent years, TLR2 has been implicated as a critical regulator of inflammation in the brain [18, 19]. While primarily a sensor for bacterial-derived pathogens, its role in disease pathogenesis is largely due to its ability to recognise protein aggregates including β-amyloid (Aβ) and α-synuclein [20, 21] along with other endogenous damage signals. Research including our own has highlighted the negative impact of TLR2 stimulation on microglial activation and the integrity of neuronal activity [13, 22–24]. In aged and AD-like experimental models, the degree of neuroinflammation and neuronal impairment is coupled to BBB disruption and infiltration of systemic immune cells [25, 26]. In particular, the contribution of macrophages to promoting the microglial activation and neuronal dysfunction associated with these conditions has been well explored [27–29].

Obesity is widely recognised among the world’s most significant population and healthcare challenges, with incidences expected to rise to 14.2% of the global population by the year 2030 [30]. It is characterised by the excessive accumulation of adipose tissue, a collection of adipocytes and associated stromal vascular fraction cells that secrete adipokines including proinflammatory cytokines, chemokines and hormones [31, 32]. Obesity is also associated with increased recruitment of proinflammatory M1 macrophages into adipose tissue further contributing to the proinflammatory milieu [33]. Together this facilitates the low-grade systemic inflammation which is characteristic of the obesogenic state [34]. Along with common comorbidities [35–37], obesity is associated with brain atrophy and cognitive decline [38–41] and increases risk of developing dementia and AD [42]. Moreover, higher levels of Aβ have been identified in obese individuals [43, 44], and diet-induced obesity markedly increases Aβ burden in animal models of AD [45, 46].

The mechanisms through which obesity influences cognitive ability are largely unknown; however, it is likely that the systemic inflammatory environment precipitates brain dysfunction [42]. Indeed, lipopolysaccharide (LPS) challenge in rats with diet-induced obesity (DIO) resulted in an enhanced and prolonged fever in addition to an increase in circulating TNF-α and IL6 [47]. Similar inflammatory changes have been reported in hippocampus of leptin receptor-deficient animals, accompanied by cognitive and behavioural deficits [48]. Integrity of the BBB is impaired by obesity [49–52], offering a route for infiltration of inflammatory cells and mediators to the CNS, similar to that reported with age and AD [26, 53, 54]. Little, however, is currently understood about how nutritional composition within an obesogenic diet, independent of weight gain, may impact on neuroinflammation. Over-consumption of saturated fatty acids (SFAs) is widely associated with obesity [55, 56]. Within the CNS, a fat-rich diet is known to induce inflammation [57], facilitate infiltration of immune cells [51, 52] and accelerate the cognitive decline associated with AD [58]. Conversely, unsaturated fats are reported for their anti-inflammatory properties [56] and are proven to convey cognitive and neuroprotective benefits [59–61]. Consumption of the monounsaturated fatty acid (MUFA)-enriched Mediterranean diet is associated with reduced risk of cognitive impairment and reduced dysfunction in experimental models of AD [62, 63], while postprandial lipoproteins isolated following a MUFA-rich meal bias microglia towards adoption of an anti-inflammatory phenotype [64]. Similarly, obesity-induced proinflammatory changes in adipose tissue are reduced by replacement of SFA for MUFA, despite equivalent weight gain [56]. Little, however, is understood about the potential impact of replacing dietary SFA for MUFA on obesity-associated neuroinflammation.

The majority of evidence implicates TLRs as the primary interface between free fatty acids and NF-κB-dependent production of proinflammatory mediators in macrophages and adipocytes [65–67]. Indeed, the SFA lauric acid has been reported to promote both TLR4- and TLR2-mediated NF-κB and cyclooxygenase (COX)2 activation in macrophages, whereas this is mitigated in the presence of PUFA [68, 69]. As free fatty acids can access the brain, it is likely that they may act similarly in the resident cells to initiate an inflammatory response. Palmitic acid (PA), the most common SFA, has been shown to modulate the microglial response to LPS [70, 71]. In light of the prominent role of TLR2 in mediating neuroinflammation, the current study set out to determine the impact of PA priming on the responsiveness of BV2 microglia to the TLR2 agonist lipoteichoic acid (LTA). As the anti-inflammatory effects of MUFA have also been reported, we evaluated whether the microglial response to LTA may be differently modulated by priming with oleic acid (OA). We further assessed whether these inflammatory effects are reflected in changes in hippocampal and cortical tissue from obese mice, following an obesogenic diet rich in either SFA or MUFA. Recognising the potential impact of systemic immune mediators in the central inflammation associated with obesity, we compared our findings in microglia to those from PA- and OA-primed macrophages. Furthermore, to model the effects of macrophage infiltration to the CNS under obesogenic conditions, we assessed their activation following exposure to soluble brain extract from chronically SFA- and MUFA-fed mice.

## Materials and Methods

### *Diet-Induced Obesity (DIO) Model*

Male C57BL/6 mice (Harlan, UK) were randomly assigned to three feeding groups: a diet rich with SFA, in particular PA (45% total kCal from palm oil) and a diet rich in MUFA, in particular OA (45% total kCal from oleic sunflower oil) or a normal, micronutrient-matched, low-fat ‘chow’ diet (10% total kCal from palm oil/oleic acid combination; Research Diets, USA). Animals were group-housed and weight-matched at baseline prior to starting the diets aged 6–8 weeks, and fed ad libitum for a total of 12 weeks. Food intake, body weights and fat-pad weights were monitored to confirm obesogenic phenotype as previously reported [56]. Animals were euthanised by cervical dislocation under isoflurane anaesthetic. Following decapitation, brains were rapidly removed and placed on ice-cold phosphate buffered saline (PBS; Thermo Scientific, UK). Hippocampal and temporal/parietal cortical tissues were isolated and blunt dissected. Tissue samples were flash frozen in liquid nitrogen and stored at − 80 °C for later use. All animal-based experiments were carried out at the UCD Biomedical Facility, following approval of the UCD Animal Ethics Committee and under licence from the Health Products Regulatory Authority of Ireland.

### Preparation of Brain Tissue Lysate and Soluble Brain Extract

Portions of isolated cortical and hippocampal tissue (described above) were homogenised in radioimmunoprecipitation assay buffer (RIPA; containing: Tris 50 mM, NaCl 150 mM, 0.5% sodium deoxycholate, sodium dodecyl sulphate (SDS) 0.1%, Igepal 1%, pH 8.0) supplemented with protease and phosphatase inhibitor cocktails (Sigma-Aldrich, UK), using a handheld homogenisation system. Homogenised samples were centrifuged at 15,000 rpm for 10 min/4 °C. Supernatants were collected and protein quantification was conducted using the bicinchoninic acid (BCA) assay (Pierce, UK). Samples were equalised with RIPA buffer and stored at − 80 °C. Separate fractions of tissue were weighed and homogenised under aseptic conditions, in sterile Tris-buffered saline (TBS; 100 μL/0.01 g) supplemented with protease and phosphatase inhibitors [28]. The tissue suspension was centrifuged at 21,000 rpm for 1 h at 4 °C. The supernatant was isolated as soluble brain extract (SBE). Protein concentration was quantified using BCA assay, equalised to 1 mg/mL in sterile TBS and stored at − 80 °C.

### BV2 and N2a Cell Culture and Treatment

Murine BV2 microglia and N2a neuroblastoma cells were grown in Dulbecco’s modified Eagle’s medium (DMEM/F12; Lonza or Sigma-Aldrich, UK) containing heat-inactivated foetal bovine serum (FBS; 10%) and penicillin–streptomycin (100 U/ml; Gibco, UK), and maintained as previously described [17, 22]. In brief, cells were plated in 24-well culture plates at a density of 1.5 × 10^5^ cells/well and incubated overnight in a humidified environment at 37 °C/5% CO_2_. BV2 cells were primed with PA or OA (100 μM; Sigma-Aldrich) for 20 h in a final volume of 200 μL per well. PA was conjugated with fatty acid-free bovine serum albumin (BSA) and reconstituted in dimethyl sulfoxide (DMSO; Sigma-Aldrich, UK) at final concentrations of 0.004% and 0.1%, respectively. OA was pre-conjugated with BSA combined with DMSO, in respective final concentrations of 0.002% and 0.07%. Vehicle controls were carried out in DMEM containing 0.004% BSA and 0.1% DMSO. Cells were exposed to LTA (1 or 5 μg/mL) ± PA or OA for a further 4 h or 24 h, respectively. N2a cells were primed with PA, OA or vehicle as above for 6 h, followed by 18 h exposure to LTA (5 μg/mL). To promote differentiation of a neuronal-like phenotype, N2a cells were incubated and treated with DMEM containing 2% FBS. Supernatants were harvested for later analysis of cytokine and nitrite concentration. Cells were stored at − 20 °C in RIPA buffer containing protease and phosphatase inhibitor cocktails (Sigma-Aldrich, UK) as previously described [22], for subsequent analysis of protein expression.

### Preparation of Bone Marrow-Derived Macrophages

Bone marrow was isolated from the femurs and tibiae of 13-week-old naïve male C57BL/6 mice in DMEM as previously described [28]. In brief, the cell suspension was filtered through a 40 μm nylon filter and centrifuged (2,000 rpm, 3 min). The supernatant was removed and the pellet was resuspended in red blood cell lysis buffer (3 ml/1 min; containing (mM): NH_4_Cl 155; KHCO_3_ 12; EDTA 0.1) and centrifuged as above. Cells were resuspended in DMEM supplemented with filter-sterilised conditioned media from L929 cells (20%) and incubated in 75 cm^2^ culture flasks for approximately 7 days. Cells were seeded in 24-well tissue culture plates at 1.5 × 10^5^ cells/well and incubated overnight at 37 °C/5% CO_2_. BMDMs were primed with PA, OA (100 μM) or vehicle for 24 h prior to LTA exposure (5 μg/mL) for 24 h. In a separate set of experiments, BMDMs were exposed to media containing SBE from DIO animals at a final protein concentration of 0.25 mg/ml (24 h). Supernatants and cell protein lysates were harvested as above and stored at − 20 °C.

### Determination of Supernatant Cytokine and Nitrite Concentration

Supernatant concentration of TNF-α and interleukin (IL)-6 was determined by enzyme-linked immunosorbent assay (ELISA), according to the manufacturer’s guidelines (Biolegend, UK). Concentration of nitrite was measured in cell supernatants using the Griess assay (Sigma-Aldrich, UK) as previously described [17, 22].

### Western Immunoblot Analysis

Protein lysates from BV2 cells, N2a cells, BMDMs and brain tissue were harvested in RIPA buffer, supplemented with protease and phosphatase inhibitor cocktails (Sigma-Aldrich, UK). Proteins (5, 10 or 20 µg) were separated using SDS-PAGE, transferred to nitrocellulose membranes and blocked in 5% semi-skimmed milk prior to overnight incubation with antibodies against iNOS (1:1,000; BD Biosciences, UK), nNOS (1:500 or 1: 1000; Cell Signaling, UK), COX2 (1:1000), PSD-99 (1:1000; Santa Cruz, USA), drebrin (1:1000; Santa Cruz, USA), synaptophysin (1:1000; Santa Cruz, USA) and β-actin (1:2,000; Santa Cruz, USA). Membranes were washed and incubated with DyLightTM 680/800 fluorescent anti-mouse or anti-rabbit secondary antibodies (Thermo Scientific, UK). Fluorescent immunoreactive bands were visualised using the LI-COR Odyssey and quantified with Image Studio Lite software.

### Statistical Analysis

Statistical comparisons were made using one-way analysis of variance (ANOVA), followed by post hoc Newman-Keuls analysis to examine the effects of a single variable between multiple groups. To assess the effects of two independent variables (e.g. LTA and fatty acid) and interactions between these effects, comparisons were made using two-way ANOVA followed by Bonferroni post-tests. All graphs and statistical analysis were carried out using GraphPad Prism 5 software. Statistical significance is represented as *p < 0.05, **p < 0.01 and ***p < 0.001 w.r.t. vehicle control and ^#^*p* < 0.05, ^##^*p* < 0.01 and ^###^*p* < 0.001 w.r.t. LTA-stimulated, unless otherwise stated in the figure legend.

## Results

### Pre-exposure of BV2 Microglial Cells to PA Attenuates LTA-induced TNF-α, but not IL-6 Secretion

**Fig. 1:**
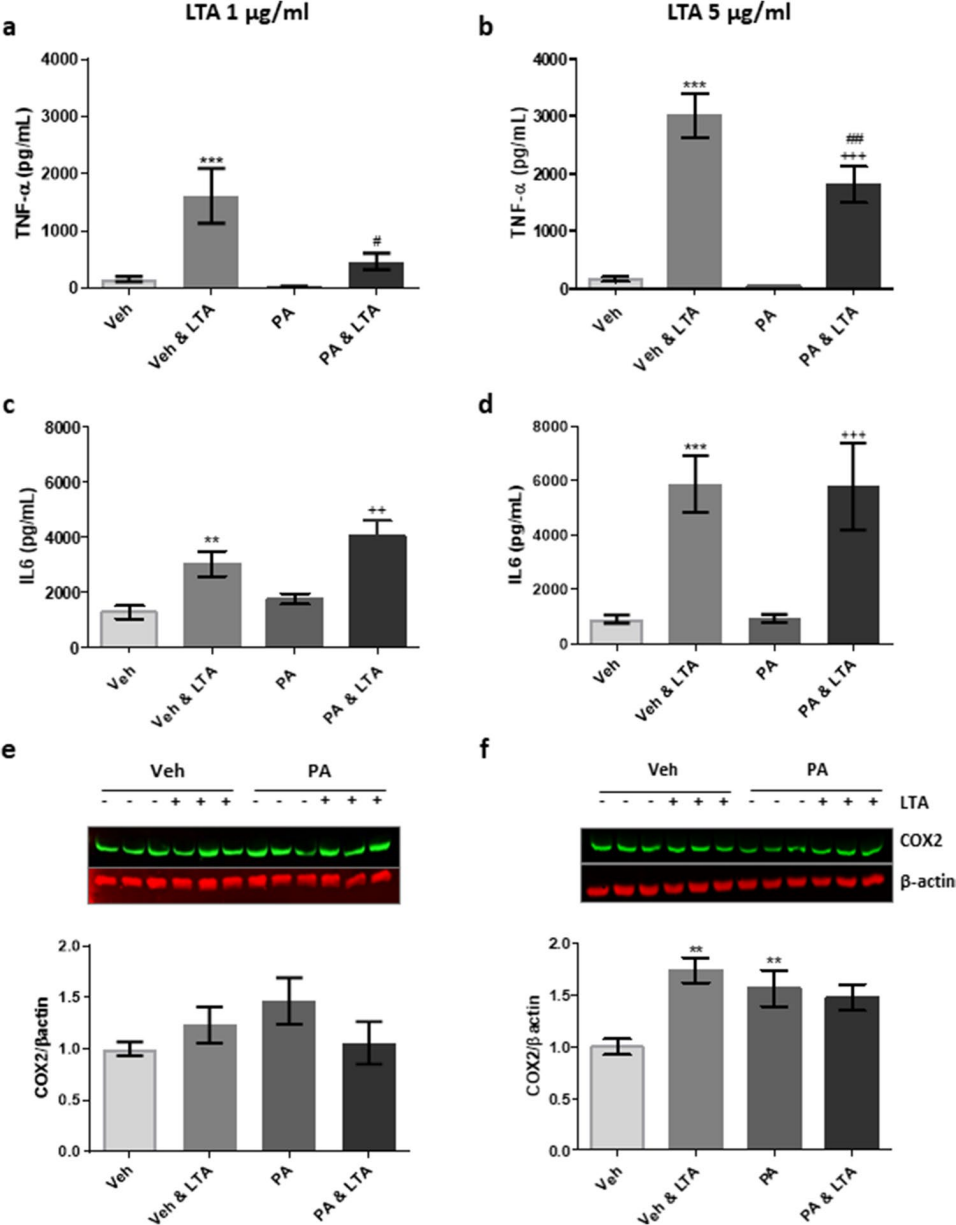
Palmitic acid attenuates LTA-induced TNF-α release from microglia. BV2 cells were exposed to palmitic acid (PA; 100 μM) or vehicle control (Veh) for 24 h, and stimulated with LTA (1 or 5 μg/mL) during the final 4 h. Supernatant concentrations of TNF-α (**a**, **b**) and IL-6 (**c**, **d**) were assessed using ELISA (*n* = 9–24 replicates). Western immunoblot was used to determine the expression of COX2 in cell lysates (**e**, **f**), relative to the expression of β-actin (*n* = 9–14 replicates from 3–6 independent experiments). Data is presented as mean ± SEM. **p < 0.01, ***p < 0.0001, compared to Veh; ^++^*p* < 0.01, ^+++^*p* < 0.0001, compared with PA; ^#^*p* < 0.05, ^##^*p* < 0.01, compared with Veh + LTA; two-way ANOVA followed by Bonferroni and Newman-Keuls analysis. Inserts illustrate representative immunoreactive bands for COX2 and β-actin (triplicate samples)

Having previously evaluated the impact of the TLR2 agonist LTA on BV2 cell activation [22], we used this as a model to examine the effect of PA on microglial cytokine production. Pre-exposure of BV2 cells to PA (100 µM) significantly attenuated supernatant concentration of TNF-α in response to both low (1 µg/ml; Fig. 1a) and high (5 µg/ml; Fig. 1b) LTA concentrations. That withstanding, pre-exposure of BV2 cells to PA did not attenuate IL-6 secretion in response to LTA at either concentration (Fig. 1c, d). Cytokine expression was accompanied by a small increase in cellular expression of COX2, which reached significance following application of 5 µg/mL LTA, when compared to controls (Fig. 1e, f). While PA alone also appeared to marginally promote COX2 expression, this did not influence the LTA-induced change (Fig. 1e, f).

### Incubation with PA Enhances LTA-Induced NO Production from BV2 Microglia

**Fig. 2:**
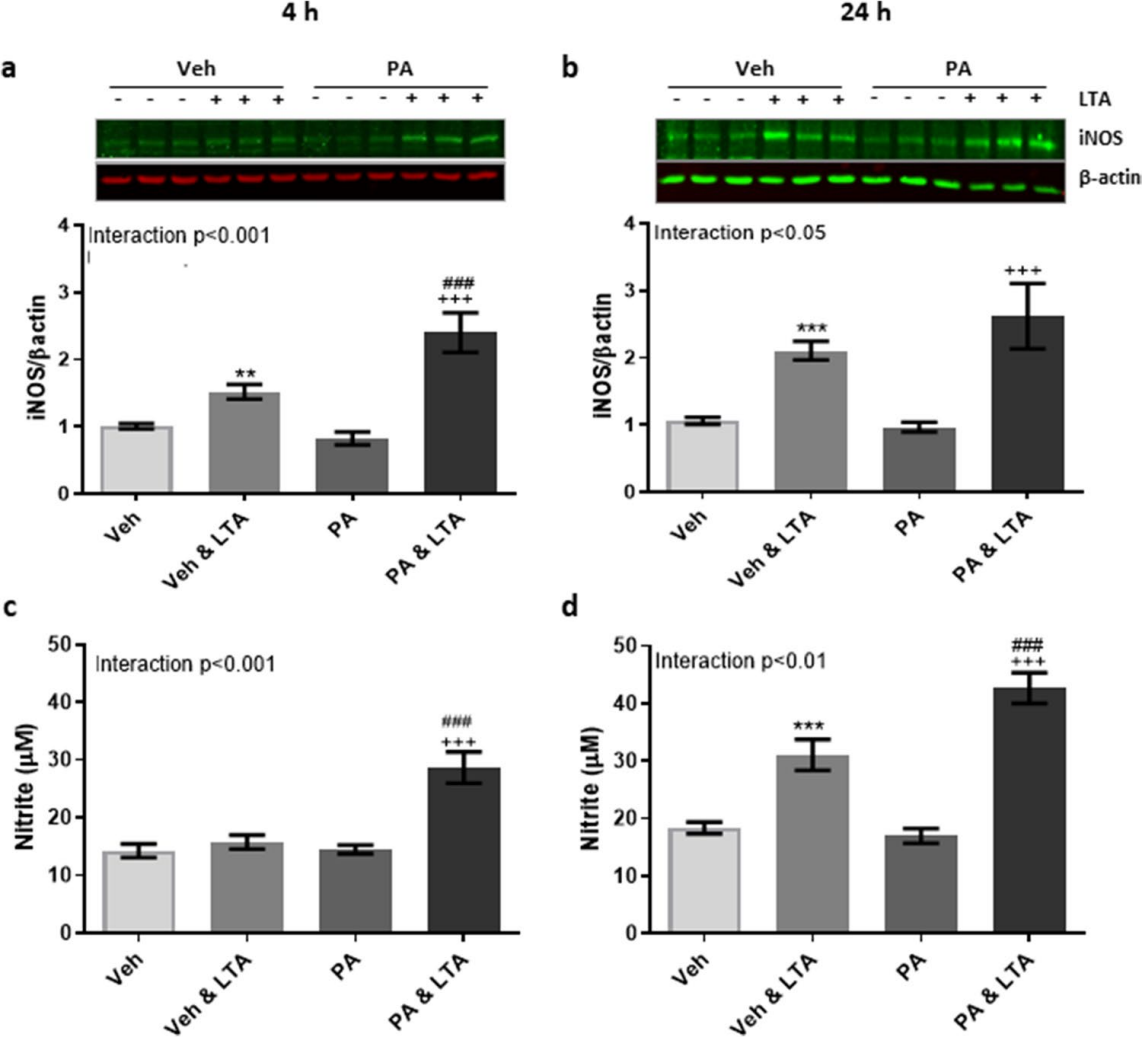
Palmitic acid promotes nitric oxide production from LTA-stimulated microglia. BV2 cells were exposed to palmitic acid (PA; 100 μM) or vehicle control (Veh) for 24 h, and stimulated with LTA (5 μg/mL) during the final 4 h, or for a further 24 h. Expression of iNOS (**a**, **b**) and supernatant concentration of nitrite (**c**, **d**) were examined. Data is presented as mean ± SEM (*n* = 6–45 replicates, from 3–12 independent experiments). **p < 0.01, ***p < 0.0001, compared to vehicle control; ^+++^*p* < 0.0001, compared with PA; ^###^*p* < 0.001, compared with Veh + LTA. Interactions based on two-way ANOVA, followed by Bonferroni post-tests. Inserts illustrate representative immunoreactive bands for iNOS and β-actin (**a**, **b**; triplicate samples)

The production of nitric oxide (NO) has become a critical determinant of microglial activation, at least under experimental conditions [72]. To evaluate its contribution to fatty acid-related changes, expression of inducible nitric oxide synthase (iNOS) and the stable NO metabolite nitrite were evaluated in BV2 cells following priming with PA. Exposure to PA significantly enhanced LTA-induced iNOS expression following 4 h LTA stimulation (Fig. 2a) with a similar trend observed in response to 24 h stimulation (Fig. 2b). Similarly, concentration of nitrite was significantly higher in the supernatant from PA-primed cells stimulated with LTA for both 4 h (Fig. 2c) and 24 h (Fig. 2d), compared with cells exposed to LTA alone (Fig. 2c, d).

### OA Has Opposing Effects to PA on LTA-Induced Inflammatory Changes in BV2 Microglial Cells

**Fig. 3:**
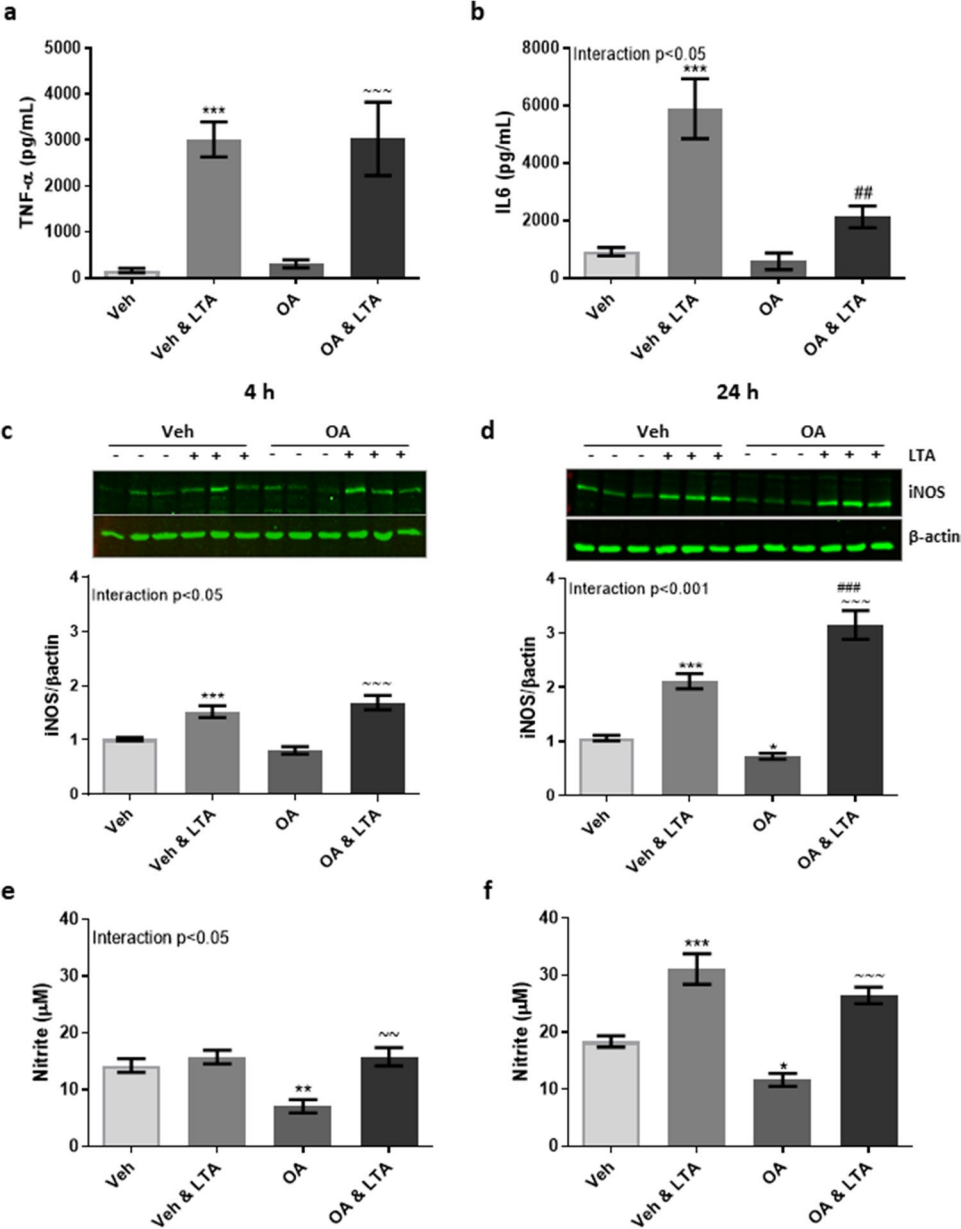
Oleic acid mitigates LTA-induced release of IL-6 and basal nitrite expression in microglia*.* BV2 cells were exposed to oleic acid (OA; 100 μM) or vehicle control (Veh) for 24 h, and stimulated with LTA (5 μg/mL) during the final 4 h, or for a further 24 h. Concentration of TNF-α (**a**) and IL-6 (**b**) was measured in the supernatant following 4 h LTA exposure (*n* = 6–45 replicates, from 3–12 independent experiments). Expression of iNOS and supernatant concentration of nitrite were examined both 4 h (**c**, **e**) and 24 h (**d**, **f**) following LTA stimulation (*n* = 9–45 replicates, from 3–12 independent experiments). Data is presented as mean ± SEM. *p < 0.05, **p < 0.01, ***p < 0.0001, compared to Veh; ~  ~  ~ *p* < 0.001, compared with OA; ^###^*p* < 0.001, compared with Veh + LTA. Interactions based on two-way ANOVA, followed by Bonferroni post-tests. Inserts illustrate representative immunoreactive bands for iNOS and β-actin (**c**, **d**; triplicate samples)

Unlike that observed in the presence of PA, pre-exposure of BV2 cells to OA (100 µM) did not alter TNF-α secretion after stimulation with LTA (5 µg/ml) for 4 h (Fig. 3a). By contrast, pre-exposure to OA significantly reduced IL-6 secretion in LTA-stimulated cells (Fig. 3b). Pre-incubation of BV2 cells with OA enhanced LTA-induced iNOS expression, which reached significance following 24 h LTA exposure when compared to cells treated with LTA alone (Fig. 3d). Most interestingly however, basal expression of iNOS was significantly reduced in unstimulated cells (24 h only) following incubation with OA, when compared with vehicle-treated controls (Fig. 3d) This was further associated with a significant reduction in basal concentration of nitrite in OA-primed cells, compared with supernatants from cells exposed to the vehicle alone (Fig. 3e, f).

### PA and OA Differently Modulate Neuronal Cell Inflammation Relative to Microglial Cells

**Fig. 4:**
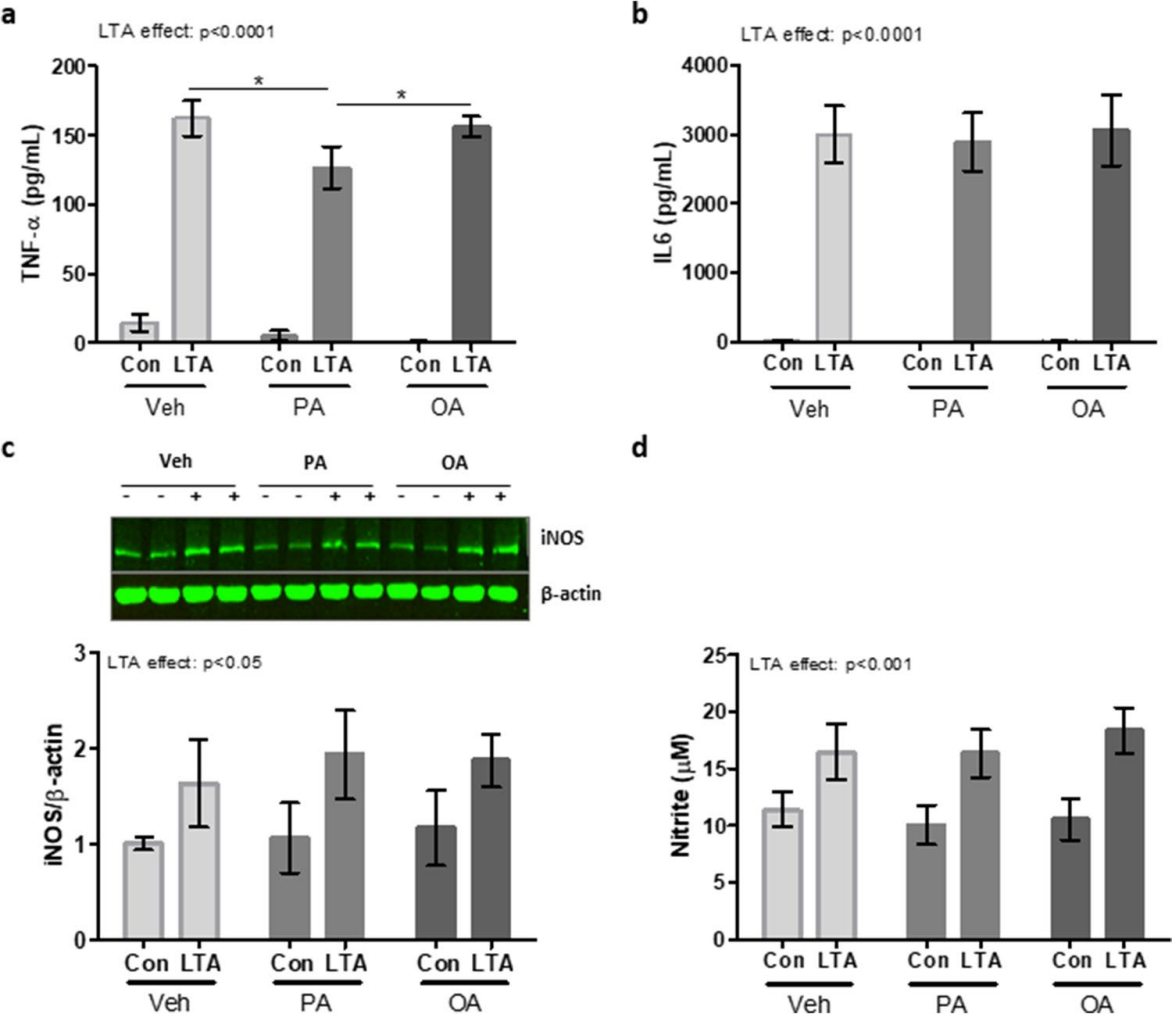
Neither priming with PA or OA alters the response to LTA in N2a neuroblastoma cells. N2a cells were incubated with PA, OA (100 μM) or vehicle control (Veh) for 6 h, prior to the inclusion of LTA (5 μg/mL) for a further 18-h period. Supernatant was assessed for concentration of TNF-α (**a**), IL-6 (**b**) and nitrite (**d**), and cell lysates were analysed for expression of iNOS (**c**) (*n* = 6–12 replicates from 3–6 independent experiments). Data are presented as mean ± SEM. *p < 0.05, **p < 0.01, ***p < 0.001; ^+^*p* < 0.05, compared to PA alone; ~  ~ *p* < 0.01, ~  ~  ~ *p* < 0.001, compared to OA alone. Comparisons were made using two-way ANOVA followed by Bonferroni analysis. Inserts illustrate representative immunoreactive bands for iNOS and β-actin (**c**; triplicate samples)

Although not primarily responsible for immune regulation in the nervous system, neurons are known to be cytokine- and NO-producing cells. Similarly to our previous finding in microglia, exposing N2a cells to PA significantly reduced LTA-induced TNF-α concentration (Fig. 4a), with no impact on IL-6 (Fig. 4b), when compared to un-primed cells. Meanwhile, incubation of N2a cells with OA did not impact the expression of either TNF-α or IL-6 in the presence of LTA, compared with un-primed cells (Fig. 4a, b). Interestingly, however, neither the expression of iNOS (Fig. 4c) or nitrite (Fig. 4d) was impacted by exposure to PA or OA, either in control or LTA-stimulated cells.

### Differential Effects of OA and PA on the Inflammatory Profile of BMDMs Stimulated with LTA

**Fig. 5:**
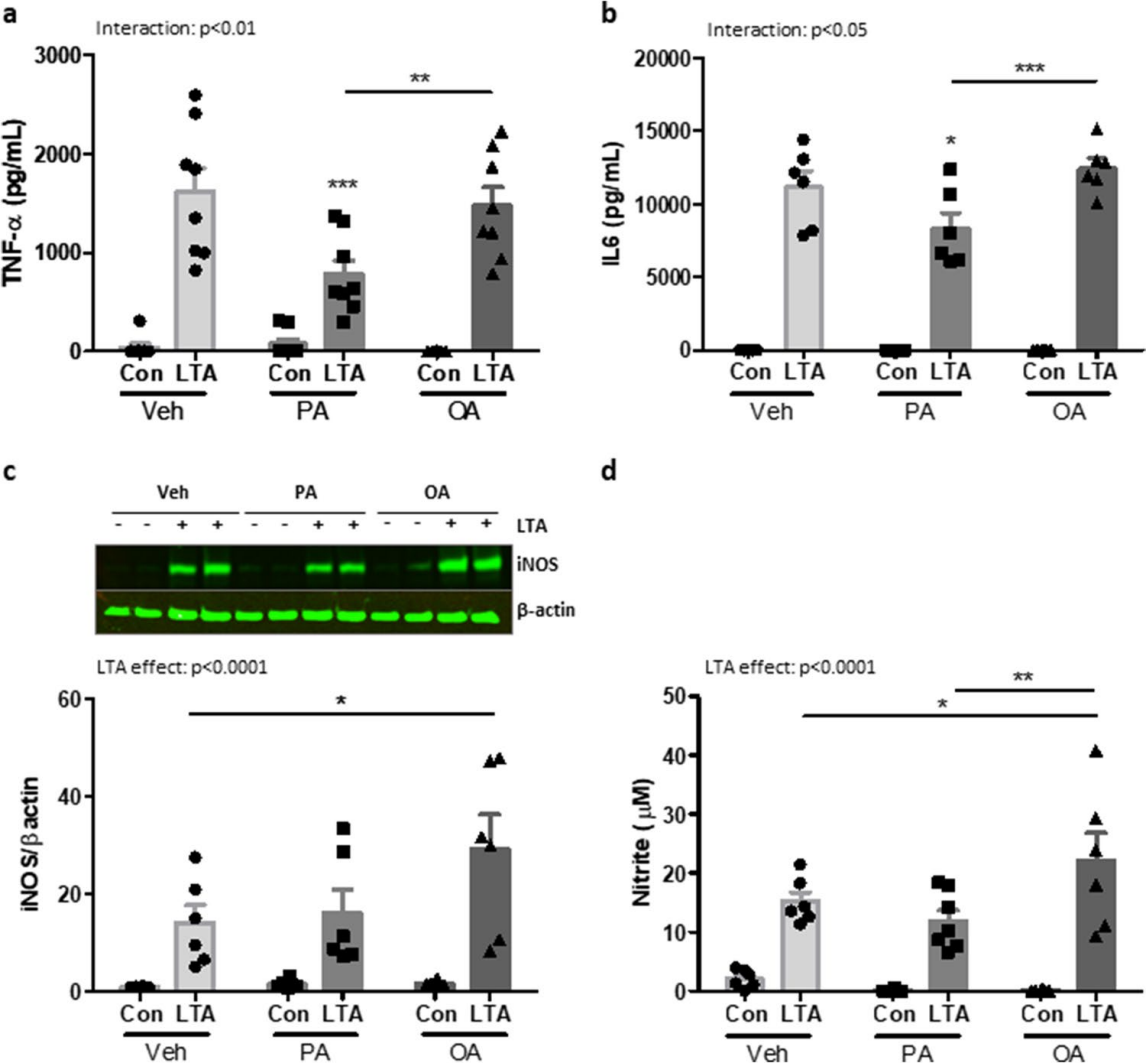
Palmitic and oleic acid differently modulates inflammatory responses in macrophages. BMDMs from C57BL/6 mice were primed with PA, OA (100 µM) or vehicle (Veh) for 24 h, followed by the subsequent application of LTA (5 µg/mL) for a further 24 h. Supernatant concentrations of TNF-α (**a**), IL-6 (**b**) and nitrite (**d**), along with cellular expression of iNOS (**c**), were measured. Data is presented as mean ± SEM (*n* = 6–8 replicates, from 3 independent experiments). Interaction and LTA effect determined by two-way ANOVA. *p < 0.05, **p < 0.01, ***p < 0.001; Bonferroni post-tests. Inserts illustrate representative immunoreactive bands for iNOS and β-actin (c; duplicate samples)

To compare the microglial response to that of their closest systemic equivalent, BMDMs prepared from naïve C57BL/6 mice were primed with PA, OA (100 µM) or vehicle for 24 h, followed by the subsequent application of LTA (5 µg/mL) for a further 24 h. Similarly to that observed in microglia and neurons, exposure to PA significantly reduced the LTA-induced release of TNF-α compared with un-primed cells (Fig. 5a). Unlike microglia and neurons, however, this effect also extended to LTA-stimulated IL-6 which was significantly attenuated in PA-primed cells compared to un-primed controls (Fig. 5b). Interestingly, prior exposure to OA had no impact on the concentration of either cytokine released in response to LTA (Fig. 5a, b). Also in contrast to microglia, exposure to PA did not influence the production of NO in LTA-stimulated BMDMs (Fig. 5c, d). However, the LTA-induced expression of both iNOS and nitrite was significantly increased in OA-primed cells compared with cells exposed to LTA alone (Fig. 5c, d).

### Chronic Exposure to Obesogenic SFA- or MUFA-enriched High-Fat Diets Did not Impact on Synaptic Density in the Cortex or Hippocampus

**Fig. 6:**
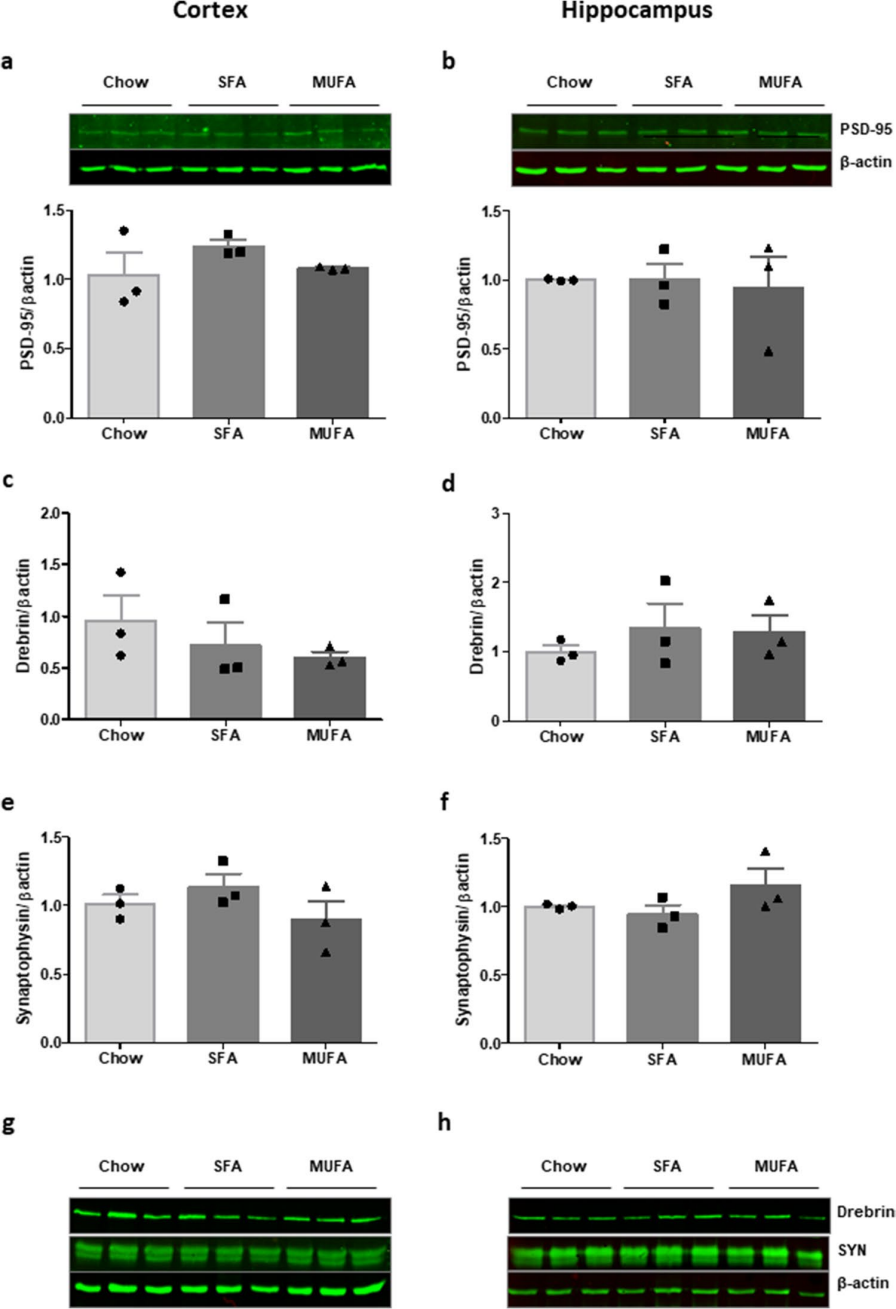
A 12-week fat-rich diet does not alter synaptic density in cortex and hippocampus. Cortical and hippocampal tissue was isolated from C57BL/6 mice, following 12-week feeding with obesogenic diets rich in palmitic acid (SFA; 45% total kCal) or oleic acid (MUFA; 45% total kCal), or a non-obesogenic, nutrient-matched control diet (chow; 10% total kCal from fat). Protein lysates were assessed for the expression of PSD-95, drebrin and synaptophysin (SYN) in cortex (**a**, **c**, **e**, **g**) and hippocampus (**b**, **d**, **f**, **h**), respectively. *N* = 3 animals per group. Data is presented as mean ± SEM overlaid with individual data points. Statistical differences were assessed using one-way ANOVA. Inserts illustrate representative immunoreactive bands for PSD-95, drebrin, SYN and β-actin (triplicate samples)

We further sought to examine how chronic exposure to SFA- or MUFA-enriched HFDs in vivo affects expression of markers of synaptic integrity within the cortical and hippocampal regions of the brain. Both HFDs induced significant weight gain, insulin resistance and hypercholesterolemia as previously described [56]. No significant changes in expression of PSD-95 (Fig. 6a, b), drebrin (Fig. 6c, d, g, h) and synaptophysin (Fig. 6e, f, g, h) were observed following SFA- or MUFA-rich diets relative to tissue from non-obese controls in either brain region.

### Chronic Exposure to MUFA- and SFA-Enriched HFDs Differently Modulates Nitrite Concentration in the Brain

**Fig. 7:**
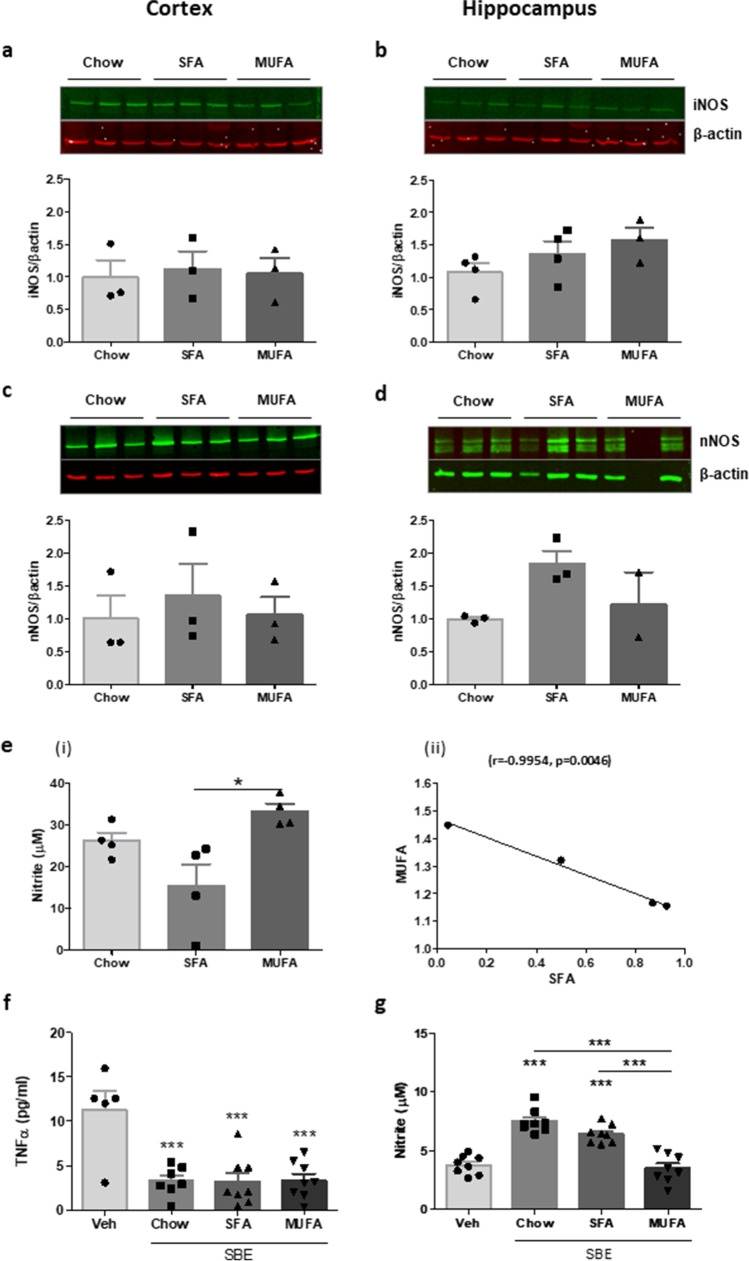
Expression of NO expression is differently regulated in brain extract from SFA- and MUFA-fed animals. Cortical and hippocampal tissue was isolated from high-fat-induced obese animals (SFA, MUFA) and non-obese controls (chow). Protein lysates were assessed for the expression of iNOS and nNOS from cortex (**a**, **c**) and hippocampus (**b**, **d**), respectively. Soluble brain extract (SBE) prepared from cortical tissue of DIO mice was assessed for expression of nitrite (**e** (i)). Correlations were made between concentration of nitrite (**e** (ii)) measured in SBE from SFA versus MUFA fed animals (*N* = 3–4 animals per group). BMDMs from naïve animals were incubated with SBE (0.25 mg/mL total protein) for 24 h. Supernatant expression of TNF-α (**f**) and nitrite (**g**) was determined. Statistical comparisons were made using one-way ANOVA, followed by Neuman-Keuls analysis. *p < 0.05, ***p < 0.001 (*n* = 5–8 replicates, from 3 independent experiments). Data is presented as mean ± SEM. Inserts illustrate representative immunoreactive bands for iNOS (**a**, **b**), nNOS (**c**, **d**) and β-actin (**a**–**d**, triplicate samples)

Recognising the dual inflammatory and regulatory role of NO in the brain, we also assessed expression of iNOS (Fig. 7a, b) and nNOS (Fig. 7c, d). Interestingly, despite our previous observations in microglia, neither HFD induced significant alterations in either iNOS or nNOS when compared to tissue from low fat-fed controls. A portion of cortical tissue was used to prepare soluble brain extract (SBE), which was assessed for expression of nitrite (Fig. 7e). Although also not robustly different from chow-fed controls, nitrite concentration was significantly lower in SBE from SFA-fed animals compared with those on a MUFA-rich diet (Fig. 7e (i)). Indeed, further analysis revealed an inverse relationship in nitrite concentration SBE from MUFA- and SFA-fed mice (expressed relative to expression in SBE from chow-fed animals; Fig. 7e (ii)). We have previously demonstrated the influence of brain-derived inflammatory proteins on macrophage activation [28]. To model the impact on macrophages which infiltrate the brain parenchyma under obesogenic conditions, we co-incubated SBE of equal total protein concentration with cultured BMDMs prepared from naïve control mice. Independently of diet composition, SBE from all groups suppressed basal secretion of TNF-α from BMDMs when compared to naïve cells (Fig. 7f). However, nitrite expression was significantly higher in BMDMs exposed to SBE from chow- and SFA-fed animals compared with cells incubated with SBE from the MUFA-fed group (Fig. 7g).

## Discussion

Obesity is known to promote the risk of brain dysfunction and cognitive decline associated with neurodegenerative states [40, 42]. However, the exact mechanisms connecting these disorders remain elusive. As neuroinflammation is integral to these collective pathologies, the current study sought to explore the impact the obesogenic diet on priming the inflammatory environment in the brain and further characterise the neuroinflammatory potential of individual fatty acids in vitro/ex vivo. Dietary fats, in particular SFA such as PA, are the most proinflammatory element of an obesogenic diet, and heightened levels of PA and OA have been identified in the AD brain [73]. Therefore, we have restricted our investigation to the role of dietary fat in regulating brain inflammation. The specific role for TLR2 in obesity, and separately in AD, has been well reported [74, 75], and we have previously described the influence of TLR2 agonists on inflammatory-induced neuronal dysfunction [22, 23]. Here we demonstrate a clear disparity between PA and OA on regulation of cytokine production, widely considered indicators of microglial activation. In particular, we report that acute exposure to PA exerts an inhibitory influence on LTA-induced TNF-α release. Our findings are in line with a previous report that basal secretion of TNF-α was mitigated in PA-exposed BV2 cells [71]; however, more specifically, we highlight that PA attenuates TNF-α secretion in response to LTA in both microglia and macrophages. By contrast pre-incubation with PA is known to heighten LPS-induced cytokine production in microglia [67] as well as in BMDMs [76]. When considered along with previous reports [70], our findings support the observation that saturated fat does not bias microglia towards the adopting the classical state of activation seen in systemic immune cells. Consistent with our findings in microglia, priming with PA also attenuated cytokine secretion from LTA-stimulated BMDMs. Based on these findings, we speculate that PA priming likely attenuates TLR2-induced proinflammatory signalling, while the same conditions exacerbate TLR4-induced signalling as reported previously [67, 70, 76]. We hypothesise that pre-exposure to PA-enriched diets may augment the systemic inflammatory response to TLR4 ligands, but potentially compromise host response to TLR2 ligands. However, a future study to evaluate this effect within a TLR2-deficient model would be essential to fully assess this hypothesis. Since SFA is known to promote NF-κB activity in macrophages [66, 69], along with post-translational cytokine processing [77], this may also suggest that PA and LTA converge on a common mechanism to disrupt cytokine production and secretion.

MUFA-rich diets are widely acknowledged for their benefits to cognitive function under challenging conditions [78], an effect which is largely ascribed to their anti-inflammatory capacity [63, 79]. Recent evidence also supports the regulation of microglial activation as underlying the protective effects of MUFA under obesogenic conditions [64]. Consistent with this, the current study reports that while OA did not modulate TNF-α release, we saw a downregulation of LTA-induced IL6 concentration in OA-primed BV2 cells. Strikingly, however, we demonstrate that PA and OA differently regulate sensitivity of microglia to NO production. NO is a critical regulator of neuronal function, and production from glial cells is widely associated with neurodegenerative and neuroinflammatory states [80, 81]. Along with TNF-α, the expression of iNOS has become a routine determinant of proinflammatory microglia [72]. Here we offer the confounding evidence that exposure to PA mitigates TNF-α secretion while enhancing NO production from LTA-stimulated microglia. Meanwhile, OA suppressed basal expression of NO in naïve BV2 cells, but had minimal impact upon LTA-induced NO production. These findings further highlight the complexity of fatty acids as inflammatory regulators in the brain, relative to the systemic immune system. Interestingly, supernatant expression of IL-1β was not detected in our samples (data not shown). Unlike previous observations in response to LPS, this data does not support a primary role for the inflammasome in mediating the FA-mediated modulation of LTA-induced inflammation.

Obesity associated with a fat-rich diet is known to promote region-specific microglial activation in the brain, within a 12- to 20-week feeding period [52, 82]. More recently, inflammatory indicators have been reported in hippocampus and amygdala within 3 days of a high-fat diet comprised of SFA, MUFA and PUFA combined [83]. Heightened PA and OA levels have been identified in the brain under neurodegenerative conditions [73, 84]. In light of the prominent microglial regulation we observed following acute application of these dietary fats in vitro, we sought to examine whether chronic exposure to PA and OA in isolation would similarly impact inflammatory processes the brain in the absence of a subsequent inflammatory challenge. Despite their obesogenic phenotype [56], we determined that a 12-week diet comprised of 45% total kCal from either SFA or MUFA did not disrupt the expression of synaptic proteins in either cortical or hippocampal tissue. While more extensive analysis is required to fully evaluate the potential impact of these diets on neuronal morphology and synaptic integrity, our limited evaluation did not support gross alteration in synaptic density. Moreover, although we identified some dysregulation in NO expression in brains of SFA- relative to MUFA-fed animals, these changes were not indicative of robust microglial activation. These findings indicate that the 12-week feeding period with either high-SFA or -MUFA in isolation was insufficient to drive marked pathological consequences within the brain. However, we also recognise that the analysis within the current study was limited to specific hippocampal and cortical brain regions, and therefore we cannot conclude that a 12-week obesogenic diet rich in SFA or MUFA in isolation does not result in more region-specific effects. Exposure to higher concentration of a multi-fat diet as demonstrated by Lainez and colleagues [52] and Butler and colleagues [83], and/or longer duration of feeding such as employed by Jeon et al. [82], is likely necessary to see significant deterioration in integrity of brain tissues concomitant with inflammatory changes.

An important outcome from the current study is the caution of examining cellular responses in isolation. This is particularly true for obesity-related inflammation, given the multi-cellular nature of the condition. Our in vitro examination indicated that the inflammatory changes in the brain in response to dietary fats are likely restricted to microglia, and moreover are mitigated by neuronal-derived mediators. Despite responding similarly to TLR agonists, neurons are thought to utilise different downstream mechanisms to process these signals [18], which likely accounts for their lack of responsiveness to fatty acids in our study. However, the bi-directional communication that exists between microglia and neurons includes an ever‐growing list of molecules known to either directly or indirectly downregulate microglial function [85, 86]. Plausibly therefore, this interaction is a contributing factor to the paucity of inflammatory changes we identified in the brain following a 12-week 45% high-fat diet, and the preservation of synaptic integrity.

The infiltration of peripheral macrophages into the CNS is thought to contribute significantly to the neuroinflammatory consequences of obesity [49, 52]. To model this interaction in vitro, we incubated macrophages with soluble factors isolated from the brain of DIO animals [28]. We found that TNF-α secretion from macrophages was similarly supressed by exposure brain extract from each feeding group, independently of diet composition or obesity. This perhaps illustrates a similar response of macrophages to the inhibitory influence of neuronal-derived factors. Surprisingly, while exposure to brain extract from chow- and SFA-fed mice enhanced NO production in macrophages, this was not evident when cells were incubated with extract from MUFA-fed mice. A consistent finding throughout the study has been enhanced production of NO, particularly in response to MUFA. Under inflammatory conditions, including TLR stimulation, NO is also an essential component of the host-defence response [87]. However, its over-production promotes cellular toxicity and accordingly has been widely implicated in disease pathogenesis [80]. A previous exploration revealed that PA and OA induce a concentration-dependent increase in NO production from LPS-stimulated macrophages [88], while a subsequent study reported that an acute 1 h pre-exposure to OA had the opposing effect on LPS-induced NO in microglia [89]. Based on our in vitro analysis of direct OA exposure and ex vivo brain extract from MUFA-fed animals, we can speculate that excess NO production in the brain in response to a MUFA-rich diet is likely driven by infiltrating macrophages and not microglia. Taken together, this evidence points towards a potential difference in the response to OA following post-prandial and chronic dietary exposure [64]. It is also tempting to speculate that disruption in the balance of NO production may therefore account for some of the apparently conflicting outcomes of the healthy ‘Mediterranean diet’ compared with the MUFA-rich obesogenic diet.

The current study focused on investigating the role of dietary fats as inflammatory regulators. Our findings reveal that exposure to fatty acids may determine the response of microglia to subsequent inflammatory challenge by a TLR2-targetting stimulus, which may have a critical impact on neurodegenerative disease processes. We further highlight NO as an important mediator of fatty acid-related sensitivity to neuroinflammation, under acute and chronic conditions. Moreover, the current findings support the proposal that the influence of a HFD on the brain is likely to be sensitive to both the total fat content and diet duration. However, we also recognise that the obese state is associated with the over-consumption of a combination of nutrients, contributing to intracellular stress and activation of an immune response [31]. As fats are rarely consumed in isolation as part of an obesogenic diet, we must also consider the influence of secondary metabolic perturbations resulting from sugar and cholesterol intake which are known to impact negatively on cognition [90], and act as primary risk factors for AD [91]. Replacing SFA for MUFA within the obesogenic diet may indeed convey some protection against cognitive decline. However, the current findings suggest that this will likely be coupled with mitigation of obesity-associated comorbidities rather than a direct influence on inflammatory processes in the brain.

## Data Availability

The datasets generated during and/or analysed during the current study are available from the corresponding author upon reasonable request.
